# The reciprocal ledge closing wedge osteotomy for post traumatic coxa vara

**DOI:** 10.1007/s11751-011-0120-5

**Published:** 2011-09-09

**Authors:** Shabir Ahmed Dhar, Tahir Ahmed Dar, Asif Sultan, Abdul Rouf Malik, Mohammed Ramzan Mir

**Affiliations:** Government Hospital for Bone and Joint Surgery, Bagat Barzullah, Srinagar Kashmir, 190005 India

**Keywords:** Reciprocating ledge, Closing wedge

## Abstract

To report a proximal femoral osteotomy with retention of bone ledges in a reciprocal position to increase bone contact and stability. The method was applied to 5 patients over a 3-year period. All patients had coxa vara. The average length gained was 1.5 cm, and the average neck shaft angle improvement was 30°. The Harris hip score improved from an average of 63 to 82. The reciprocal ledge osteotomy is technically less demanding and also allows conversion of normal shear forces around the upper femur to stabilizing forces. This method allows easier use of the DHS implant as potential rotation about the axis of the screw is negated by the ledges and the dynamic forces.

## Introduction

Varus malunion is the most common deformity after an intertrochanteric fracture, resulting in an abductor muscle imbalance causing limp, shortening, hip, back, and knee pain.

Several types of osteotomies have been developed for the management of varus malunion. These can be divided into three main groups: Transverse opening wedge, the transverse closing wedge, and the ball and socket osteotomy [1]. In closing wedge osteotomies, it is necessary to obtain total apposition of raw surfaces. Slightest variation in this technique causes point contact between the two major fragments. This precludes the original surgical goals that include total contact between the two surfaces, prevention of rotation of the proximal fragment, and maintenance of the psoas attachment to the distal fragment.

We describe a technique wherein a modification in the closing wedge method allows point contact to be avoided while allowing the surgeon to use the dynamic hip screw for stabilization. This also allows a wider implant angle choice compared to the conventionally used fixed angle plates.

## Materials and methods

We used this osteotomy for posttraumatic coxa vara in five patients from Jan 2006 to Jan 2009. The age of these patients ranged from 25 to 58 years with a mean of 48 years. All patients had suffered from a fracture of the intertrochanteric region with 4 having simple trauma and malunion and the other one having an additional osteomalacia with coxa vara.

## Preoperative planning

In all cases, an X ray of the pelvis with both hips is taken and the neck shaft angle of the normal side calculated. The neck shaft angle of the abnormal side is also calculated. A paper tracing is made, and the tracing cut along a horizontal line through the lesser trochanter. The two sections are overlapped around an axis through the lesser trochanter. The required neck shaft angle is obtained, and the angle at the overlap is calculated. A K wire is bent at the same angle to be used as a template intraoperatively.

## Biomechanics

The intertrochanteric region of the femur is acted upon by several forces. The Psoas muscle flexes abducts and externally rotates the proximal fragment. The adductors acting on the distal fragment cause shear forces as the proximal fragment is abducted by the gluteus minimus and maximus. Any osteotomy in this region also has to consider the 1,200 lb/sq inch forces in the sub-trochanteric region [2]. The idea of an osteotomy through the lesser trochanters envisages the use of the proximal forces to advantage as the proximal fragment with a ledge gets locked beneath the distal fragment. The anterior ledge gives additional stability.

## Surgical technique

This method has been applied to varus intertrochanteric malunion in our hospital. The prerequisite for the procedure is the availability of 60° of flexion at the hip. On a traction table under image intensifier control, the trochanteric region and the proximal 10–13 cm of the femur are exposed by a lateral incision. The vastus lateralis is reflected medially. A Kirschner wire bent to an appropriate angle as calculated preoperatively is used to mark the wedge.

**Fig. 1:**
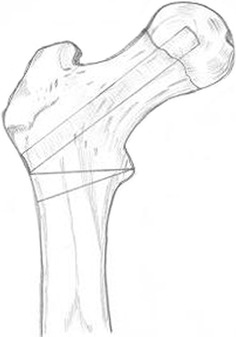
Shows the relationship of the osteotomy cuts with the richard screw track

**Fig. 2:**
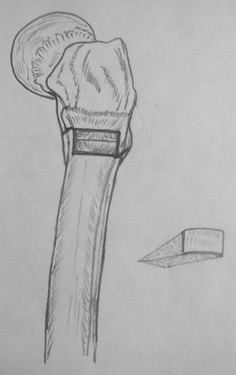
The wedge is taken out and the ledges are retained and reciprocal cuts made

**Fig. 3:**
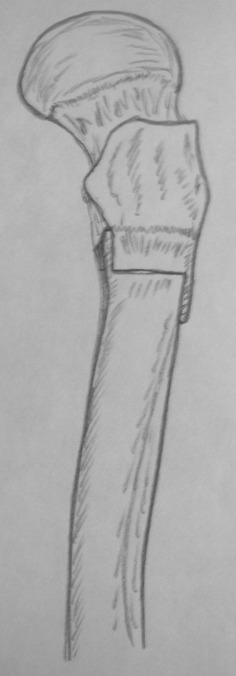
Once the wedge is closed, the ledges overlap reciprocally

The apex of the wedge intersects the lesser trochanter at the junction of its upper one-third and lower two-thirds. The upper cut of the osteotomy is made horizontally. The cortices of the femur are marked as per the template. A derotation line is marked on the lateral aspect of the bone. At a point that is half the diameter of the barrel reamer proximal to the proximal mark, a guide wire is inserted into the femoral neck ensuring its central location in the anteroposterior and lateral planes with an image intensifier (Fig. 1). The angle that the guide wire subtends with the lateral cortex is calculated with an angle guide. This angle is added to the osteotomy angle and the corresponding barrel plate kept ready. The reaming over the guide wire is completed, and a dynamic hip screw inserted. The osteotomy is performed with a saw cutting through lateral cortex at both the marks. The wedge of bone is removed leaving the anterior and posterior cortex intact. The posterior cortex is osteotomized at its inferior extent, whereas the anterior cortex is osteotomized at the proximal extent in a reciprocal manner (Fig. 2). The lower fragment is externally rotated, and the iliopsoas is completely detached from the remnant of the lesser trochanter. The barrel plate is inserted. The angle between the plate and the femur and the angle between osteotomy cuts should be equal. The femoral shaft is approximated to the plate that closes the osteotomy and overlaps the ledges posteriorly as well as anteriorly. Cortical screws are inserted to fix the plate in a compression mode. The wound is closed over suction drains (Fig. 3).

**Fig. 4:**
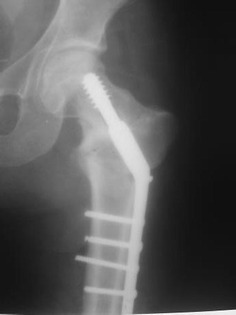
Radiograph showing the final picture in a patient operated for coxa vara

Postoperative treatment includes walking with crutches from postoperative day 1. The patient starts range of motion exercises at the same time. The patients progress to partial weight bearing at 5 weeks. Full weight bearing is encouraged after radiographic union is obtained (Fig. 4).

## Results

We operated five cases of intertrochanteric and basicervical varus malunion. Average length gained was 1.5 cm, and the average neck shaft angle improvement was 30°. The Harris hip score improved from an average of 63 to 82. Average preoperative hip score 60–65, and average postoperative hip score 80–89.

The only problem that is encountered with this procedure is that the hip screw is not always centrally placed as its entry point is subservient to the proximal cut of the osteotomy. However, we did not encounter any cut outs, and all patients had united by 8 weeks.

## Discussion

The versatility of proximal femoral osteotomy technique is attested to by the fact that a number of conditions including unstable trochanteric fractures, femoral neck nonunion, and abductor dysfunction can be treated by this method [3].

The proximal femur has several dynamic forces acting on it. The iliopsoas, the gluteus medius, and minimus exert a flexion, abduction, and external rotation force. Any osteotomy above the lesser trochanteric area relies entirely on the implant to negate these forces.

Open wedge osteotomies have the advantage of gaining length and being adjustable if plaster of Paris is used. However, more commonly, these osteotomies need stabilization with a fixation device and interposition of a bone graft or a spacing agent [4–6]. Paccola and Fogagnolo enumerated insufficient or excessive axial correction, loss of reduction, delayed union, and pseudoarthrosis as complications of open-wedge osteotomy [7]. Dome osteotomy maximizes the area of contact and is more effective for acute deformity correction [8]. Maquet reported that the dome osteotomy allowed more accuracy and adjustability of correction, while highlighting the technical difficulty, intraarticular fracture, and scarring of the extensor mechanism as complications [9].

Hankemeier et al. reported that in a dome osteotomy, correction can be achieved without secondary translation [10]. Interestingly, they subclassified the dome osteotomy into closing, neutral, and opening corrections, thereby bringing the concept of total bone contact into question.

The reciprocating ledges help in ensuring contact even when a slight error of a few degrees is made. Also, the dynamic hip screw allows controlled collapse allowing earlier union. This is important in case there is a slight translation due to a few degrees of discrepancy between the angle of the osteotomy and the barrel plate. The resultant medialization is helped by the controlled collapse. The fixed double angle plate often causes nonunion in comparison [11].

Schonfeld et al. have reported a series of four cases where they used the DHS. They reported good results citing familiarity with this instrumentation as an advantage [12].

## Conclusion

The reciprocal ledge osteotomy that is technically less demanding and also allows conversion of normal shear forces around the upper femur to stabilizing forces. This method allows easier use of the DHS implant as potential rotation about the axis of the screw is negated by the ledges and the dynamic forces.
